# Landscape characteristics influence helminth infestations in a peri-domestic rodent - implications for possible zoonotic disease

**DOI:** 10.1186/1756-3305-7-393

**Published:** 2014-08-26

**Authors:** Götz Froeschke, Sonja Matthee

**Affiliations:** Department of Conservation Ecology and Entomology, Stellenbosch University, Private Bag X1, Stellenbosch, 7602 South Africa

**Keywords:** Landuse, Zoonotic disease, Helminth, Small mammal, South Africa

## Abstract

**Background:**

Anthropogenic habitat change often results in altered landscapes that can provide new environments where hosts, parasites and pathogens can interact. The latter can have implications for human and animal health when in close proximity to developed areas. We recorded the helminth species richness and level of infestation in the peri-domestic rodent, *Rhabdomys pumilio*, in three different human linked landscapes. The aim was, to investigate the potential of *R. pumilio* to act as a reservoir host for zoonotic helminths and to compare the effect of anthropogenic habitat change on its parasite infestation patterns.

**Methods:**

Rodents (n = 518) were trapped in natural areas (nature reserves) and in three human linked landscapes (crop, livestock and urban fragments). Gastrointestinal parasite burdens were recovered and helminths identified from each animal. Generalized linear models were applied to investigate the effect of different landscape types on helminth infestation.

**Results:**

*Rhabdomys pumilio* was the most abundant rodent species within each landscape type. Eight helminths species were recovered and overall helminth prevalence was 86.68%. Mean helminth species richness, prevalence and abundance were significantly higher in crop fragments compared to natural landscapes and overall lower for nematodes in livestock and urban areas. Cestode prevalence showed a tendency to be elevated at anthropogenic linked landscape types.

**Conclusions:**

Host parameters and parasite infestations were strongly influenced by landscape characteristics. Resource-rich landscapes (crop fragments) provide favorable conditions for helminth infestations, while landscapes that are more closely associated with humans (livestock and urban landscapes) pose a larger risk by zoonotic species.

**Electronic supplementary material:**

The online version of this article (doi:10.1186/1756-3305-7-393) contains supplementary material, which is available to authorized users.

## Background

Parasites are omnipresent in the lives of wild animals and represent a major component of biological diversity[1]. More than 50% of the known species on this planet are parasites or pathogens of some form[2] and over 60% of the known human pathogens are zoonotic[3]. A recent report by the Department for International Development, UK (2012) noted that the most important zoonoses in terms of human health impact, livestock impact, amenability to agricultural interventions, severity of diseases and emergence are of a gastrointestinal zoonotic nature. Helminths represent the most prevalent macroparasite group of endoparasites[4] and among infectious diseases helminthiases are regarded as a key issue. Helminths and especially gastrointestinal nematodes can have a large impact on human and animal health[5, 6]. In 1979, the World Health Organization (WHO) Expert Committee on Parasitic Zoonoses[7] already identified 17 nematodes, five cestodes and 12 trematodes as the cause of important human infections in which other vertebrate animal hosts play epidemiologically significant roles. Furthermore, helminths have the capacity to regulate the abundance of wild animal populations[8] and communities[9] and hence may affect the functioning of ecosystems.

The key event in emergence of most infectious diseases is a change in host-parasite relationships, resulting from changes in human demography, behaviour or social structure[10–12]. Gastrointestinal helminths of terrestrial mammals spend at least one part of their life cycle in the external environment outside their host. Habitat characteristics and environmental variables, such as humidity and temperature, are important for the survival of eggs and larvae[6, 13, 14] and hence affect the prevalence, intensity and geographic distribution of helminths[15, 16]. Similarly, vegetation structure and landscape composition are important determinants for small mammal populations[17].

During the processes of landscape fragmentation and urbanization, both wild and domestic animals and humans have the potential to experience new interactions in altered environments, which provide opportunities for parasite and pathogen exchange[18, 19]. Habitat fragments, especially those that occur within transitional boundaries, favour small-sized generalist mammal species, such as rodents, which are able to adapt to or exploit these conditions[20, 21]. Rodents represent 40% of mammalian species[22] and have been pointed out as the major reservoir of zoonoses within this class, with significant impact on public health[23, 24]. They can also transmit pathogens to domestic animals, which may act as an additional reservoir for human exposure[25]. Given the above it is quite possible that elevated disease risk might be associated with landscape characteristics that facilitate higher rodent densities through the provision of resources (food, shelter and water)[26–28] or the absence of predators[29].

To date little is known about the relationship between landscape characteristics, host dynamics and host-parasite interactions[30]. A recent study by McFarlane et al.[31] reviewed data from the Asian-Australian region and found that synanthropic mammalian hosts (mainly rodents and bats) are more commonly associated with emerging infectious diseases than other wildlife in this region. In addition, a comparative study conducted on rodents in Southeast Asia recorded that microparasite diversity (viruses, bacteria and protozoan) was positively associated with flat agriculture land (i.e. flooded, irrigated, paddy fields)[32]. Along the same lines, several studies have confirmed that anthropogenic linked habitat change facilitates helminth species richness[30, 33–39]. Although results are inconsistent and seem to be dependent on the host species[35, 38], there appears to be a positive relationship between helminth species richness and forest fragments, agriculturally used areas and human settlements[30, 33, 36].

Human linked habitat transformation often results in heterogenous landscapes with surrounding fringe areas[21]. However, land use activities and the surrounding matrix will influence the resulting effect on host and parasite assemblages. Comparative land use studies are limited and while the role of cities in human infectious disease is well established, the dynamics of urban-wildlife-pathogen interactions is largely unexplored[40]. Empirical baseline data on helminth burdens and the factors that influence parasite response to different landscape characteristics are needed to make firm predictions about the effects of anthropogenic land use on possible emerging zoonoses[41]. The Cape Floristic Region in the Western Cape Province of South Africa is recognised as a global biodiversity hot spot[42]. The region is also affected by agricultural activities and urbanisation that threaten natural habitat[43]. The objective of the study was to compare the helminth burdens in a peri-domestic rodent species, *Rhabdomys pumilio,* trapped in different human linked landscapes. *Rhabdomys pumilio* has successfully adapted to agricultural and peri-urban habitats, where it is often regarded as a pest species[44]. More importantly, the species harbours a diverse assemblage of macroparasites (ticks, mites, fleas, lice and helminths)[16, 45–57]. Some of the known ectoparasites that infest *R. pumilio* are of importance in the etiology of zoonotic diseases in humans such as plague (*Yersinia pestis*) and Crimean-Congo haemorrhagic fever[58, 59] and they may be involved in the transmission of diseases of domestic animals (e.g. tick bite fever in dogs and anaplasmosis in cattle, sheep, and goats)[53, 59]. Moreover, novel paramyxoviruses and Hepatitis C virus were recently discovered in *R. pumilio* populations in the Western Cape Province[60, 61]. To date most of the quantitative parasite studies on *R. pumilio* have focussed on ectoparasites, with very little comparable research being conducted on helminths[16, 28]. This is a concern, given the fact that poor sanitation and malnutrition, in especially rural and semi-urban areas, may predispose humans to helminth infestations[62]. A case in point is a recent survey that recorded a 55.8% infestation rate for soil-transmitted helminths in young children in the Cape Town region, South Africa[63]. The authors also reiterated the need for continuous monitoring in schools and communities in an attempt to identify the potential source of infection in this region[63]. Proactive control will only be possible once reservoir hosts and high-risk landscape are identified[41, 64].

Therefore, the specific aim of the study was to record the helminth species richness and level of infestation in the peri-domestic rodent, *Rhabdomys pumilio*, trapped in three human linked landscapes. It is predicted that hosts and parasites will have variable responses to different types of landscapes. In particular, it is predicted that landscapes that favour high rodent densities will harbour both larger helminth species numbers and higher infestation levels. It is anticipated that novel data will be recorded on the reservoir potential of *R. pumilio* for zoonotic helminths in different landscapes.

## Methods

### Study area, sample collection and parasite recovery

The study was conducted in lowland fynbos vegetation with patches of renosterveld in the Cape Floristic Region of the Western Cape Province (Table1). Rodents (*Rhabdomys pumilio*) were trapped at 16 localities. Four localities were situated in nature reserves and represent extensive natural vegetation with dense shrub cover. The other twelve localities were remnant fragments of which four were surrounded by vineyards or crop fields (referred to as crop fragments), four were in the midst of livestock farms (predominantly cattle or sheep, referred to as livestock fragments) and another four fragments were within urban areas. The crop fragments were characterized by the presence of shrub cover and vegetation that was chopped and left in situ, availability of food (e.g. seasonal wheat surrounding the fragments) and water (farm dams and natural streams). Livestock fragments predominantly consisted of grazed, open grasslands with low vegetation cover. Fragments in urban areas consisted mainly of recreational areas with shrubs and grasses surrounded by houses. The three latter fragments were exposed to dogs at variable levels, with the highest frequency associated with urban fragments.

**Table 1:** **Locality and trapping information**

Locality	Geographic location	Size [km^2^]	Sample size of ***R. pumilio***	Number of small mammal species
Natural				
Jonkershoek	33° 55′ 51.00″ S, 18° 51′ 15.98″ E	98.00	40	6
Elandsberg Nat.	33° 26′ 25.15″ S, 19° 03′ 02.30″ E	40.00	32	2
Hottentotsholland	33° 59′ 16.98″ S, 19° 04′ 46.99″ E	70.00	42	4
Helderberg	34° 03′ 24.41″ S, 18° 52′ 03.04″ E	2.54	34	4
Crop				
Zevenwacht	33° 55′ 02.96″ S, 18° 43′ 56.06″ E	1.10	43	6
Elandsberg Agr.	33° 26′ 25.15″ S, 19° 03′ 02.30″ E	0.63	26	3
De Rust	34° 10′ 27.98″ S, 19° 04′ 46.99″ E	0.90	50	3
Cordoba	34° 02′ 03.41″ S, 18° 43′ 56.06″ E	0.31	53	4
Livestock				
Elsenberg	33° 50′ 04.45″ S, 18° 51′ 02.16″ E	0.67	30	2
Wellington	33° 31′ 44.40″ S, 19° 02′ 27.49″ E	0.46	26	3
Gordons Bay	34° 08′ 48.55″ S, 18° 53′ 16.29″ E	0.13	30	1
Franschoek L.	33° 51′ 11.48″ S, 18° 58′ 20.60″ E	0.38	20	1
Urban				
Stellenbosch	33° 55′ 57.39″ S, 18° 52′ 39.79″ E	0.20	30	3
Somerset West	34° 03′ 37.36″ S, 18° 49′ 42.49″ E	0.10	24	2
Franschoek U.	33° 54′ 34.77″ S, 19° 07′ 33.16″ E	0.03	20	1
Khayelitsha	34° 02′ 49.52″ S, 18° 39′ 25.95″ E	0.10	18	2

Localities per landscape type, their coordinates, sizes and the number of sampled animals per locality.

To keep the possible effect of temporal variation to a minimum, *R. pumilio* individuals were trapped within the warm-dry period from October to December (austral spring and summer months) in 2003, 2004 (natural landscapes and crop fragments)[56], 2010 and 2011 (livestock and urban fragments). We used line transects, consisting of Sherman-like live traps ca. 10 m apart from one another, which were baited with a peanut butter and oats mixture. The number of traps used per locality ranged from 48 to 200. Transects were placed > 5 meters away from the edge and where possible, within the central area in each locality. The aim was to catch 30 adult individuals (mass ≥ 32 g)[65] at each locality per trap period but it was not everywhere possible due to low rodent densities at certain localities (Table 1). Individuals of the target species were euthanized with 2–4 ml Sodium Pentobarbitone (200 mg/kg), depending on individual weight, and non-target species were identified and released at the trap site. The project was approved by the Ethics Committee of Stellenbosch University (ref no 2006B01007 and SU-ACUM11-00004(p)) and permits issued by Cape Nature (ref no. 317/2003, 360/2003, AAA004-00221-0035). Each rodent was placed in a separate, pre-marked bag and rodents were frozen at -20°C until examination. The body weight, total length (measured from nose to tail tip), tail length, sex and reproductive state of each individual (n = 518) were recorded. The methods used for endoparasite recovery and identification are described in more detail elsewhere[16]. In short, the gastrointestinal tract of each animal was dissected and the stomach, small intestine, caecum and colon were examined separately under the stereoscopic microscope and compound microscope (Leica Microsystems GmbH, Wetzlar, Germany). Helminths were carefully removed and washed out from the mucosa, counted and identified. Reference species, taxonomic keys, scanning electron microscopy and published species descriptions (a list can be supplied by the authors) were used for identification.

### Data analysis

We calculated the species richness (number of helminth species per individual host), the prevalence (infected – not infected) and the abundance (number of individuals per helminth species and overall) per individual host. For the abundance, worm numbers were log transformed to improve normality. Cestodes were excluded from abundance analyses because a correct census could not be warranted due to disintegration in older samples. The two congeneric cestode species *Hymenolepis* (syn. *Rodentolepis*) *nana* and *Hymenolepis microstoma* were recorded. Due to low prevalence levels it was decided to combine the two species for statistical analysis. Relative host density was estimated by dividing number of trapped animals, by number of trap nights multiplied by number of traps used. In each case the standard deviation was reported with the mean. We checked our data for possible spatial structure using Moran’s *I* coefficients and correlograms implemented in Spatial Analysis in Macroecology (SAM, Version 4.0)[66]. Since no spatial autocorrelation could be found (results not shown), we applied generalized linear models (GLMs), which were fitted for the species richness, the overall and species-specific helminth prevalence and abundance. The species richness model was calculated using a Gaussian error distribution; prevalence models were calculated using a binomial error distribution and logit link function, and abundance using a quasipoisson error distribution and log link function. We started with the full model, which included as predictor variables: landscape type, relative host density, host total length (used as a surrogate for host body size), sex and the interactions of length:landscape type, relative host density:landscape type as well as year. Exploratory data analysis did not reveal any significant differences in body size between sexes in any landscape type (t-Tests, all p > 0.05) and was therefore not included. Afterwards we conducted a backward selection to find the minimal adequate model to explain our data[67]. Backward selection was performed by dropping step-by-step non-significant predictors from the model, thereby following the guidelines of model simplification as proposed by Crawley[68] and Zuur et al.[69]. The new, less complex model was compared with the previous, more complex model by testing the change in deviance for significance. If the simplification was not associated with a significant increase in deviance, the less complex model was preferred[67]. Statistical tests were performed in R (version 2.15. R Development Core Team 2012, Austria), applying the MASS package[70]. The percentage of explained deviance for each model was calculated as (null deviance – residual deviance)/null deviance.

## Results

**Figure 1:**
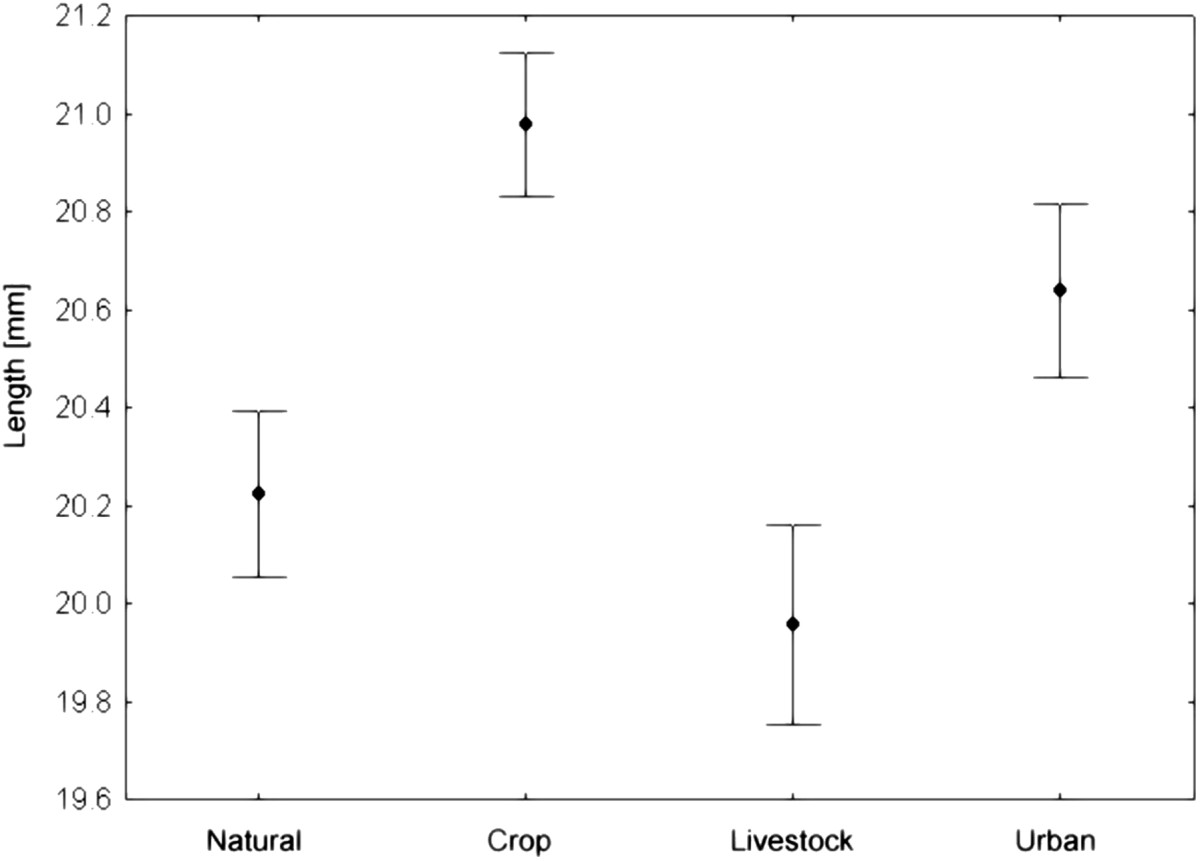
**Mean total body length [mm] (±95% CI) for** ***Rhabdomys pumilio*** **individuals trapped in different landscape types.**

**Figure 2:**
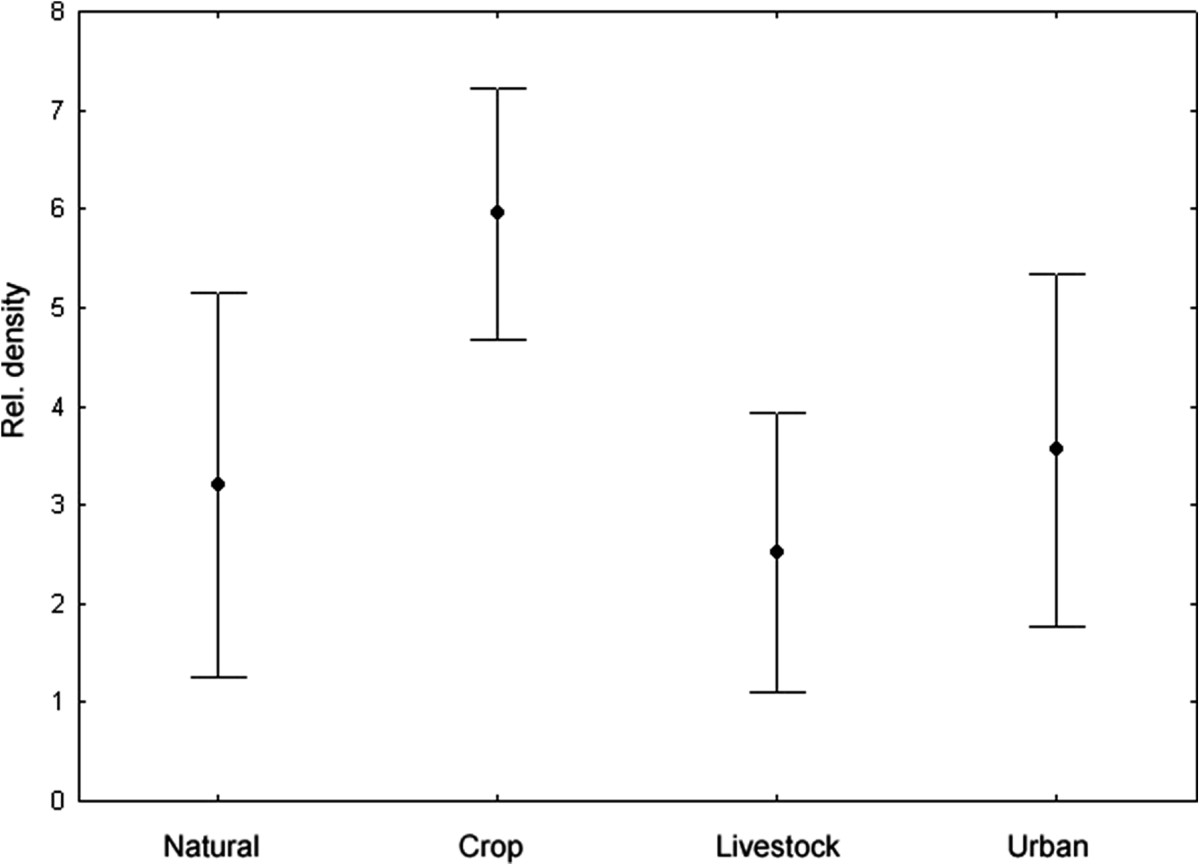
**Relative host density (±95% CI) of** ***Rhabdomys pumilio*** **in respective landscape types.**

The small mammal species richness differed between the landscape types, however *R. pumilio* was the most abundant rodent species at each. The highest species richness was recorded in natural areas and crop fragments compared to livestock- and urban fragments (Table 1). The total body length of *R. pumilio* individuals was significantly different between landscape types (ANOVA: F_3, 514_ = 7.312, p = 0.001; Figure 1) with longest individuals in crop fragments (20.98 ± 1.9 cm) and shortest animals at livestock fragments (19.95 ± 2.1 cm). Overall relative host densities of *R. pumilio* across different landscape types were not significantly different (Kruskal-Wallis: H = 0.382, p = 0.282; Figure 2) but it was highest at crop fragments (5.95 ± 2.6) and lowest at livestock fragments (2.52 ± 2.8).

**Figure 3:**
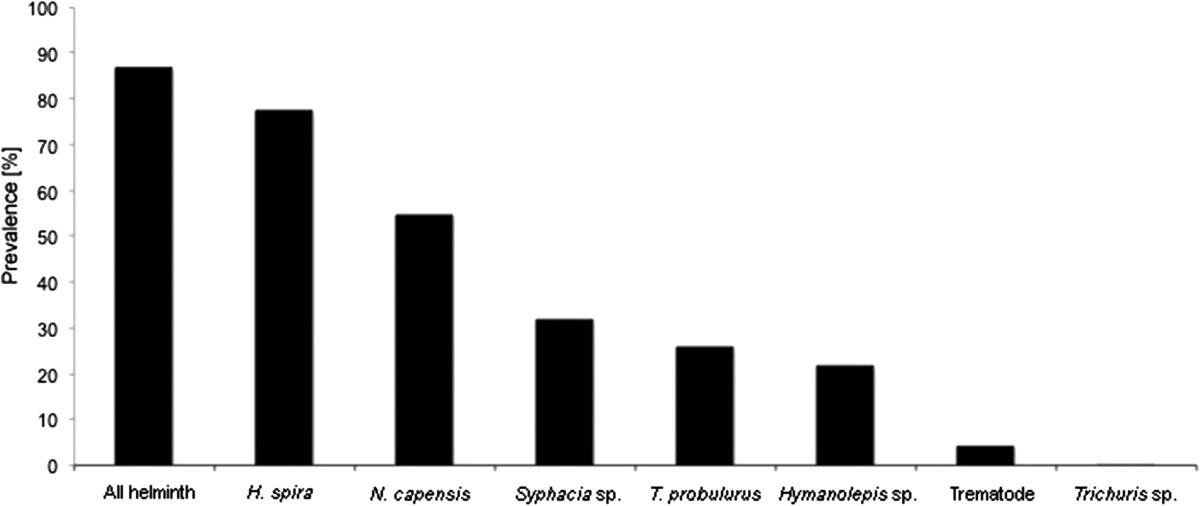
**Overall prevalence [%] of all helminths together and each helminth species respectively.**

**Figure 4:**
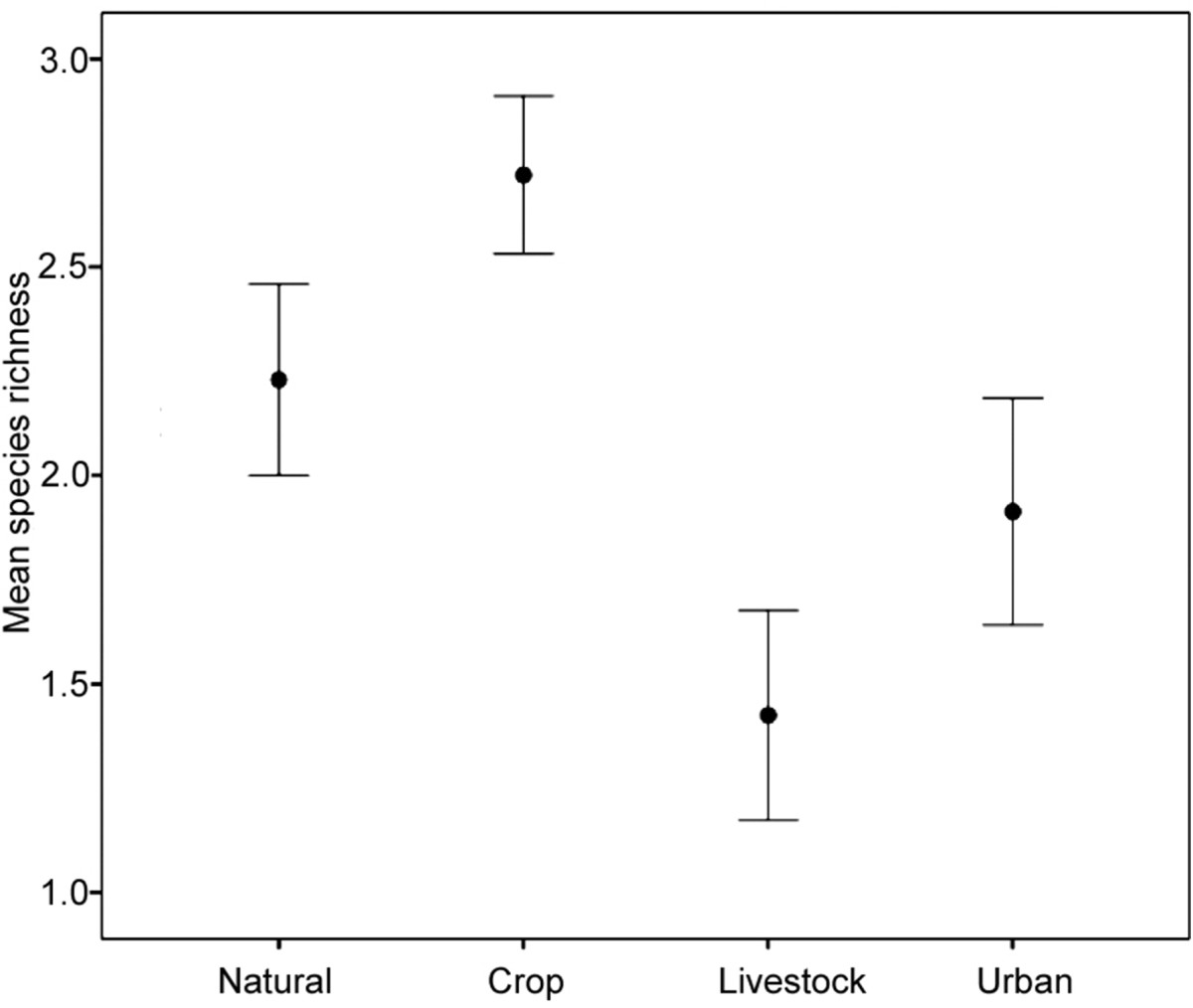
**Mean helminth species richness in** ***Rhabdomys pumilio*** **per landscape type.**

**Figure 5:**
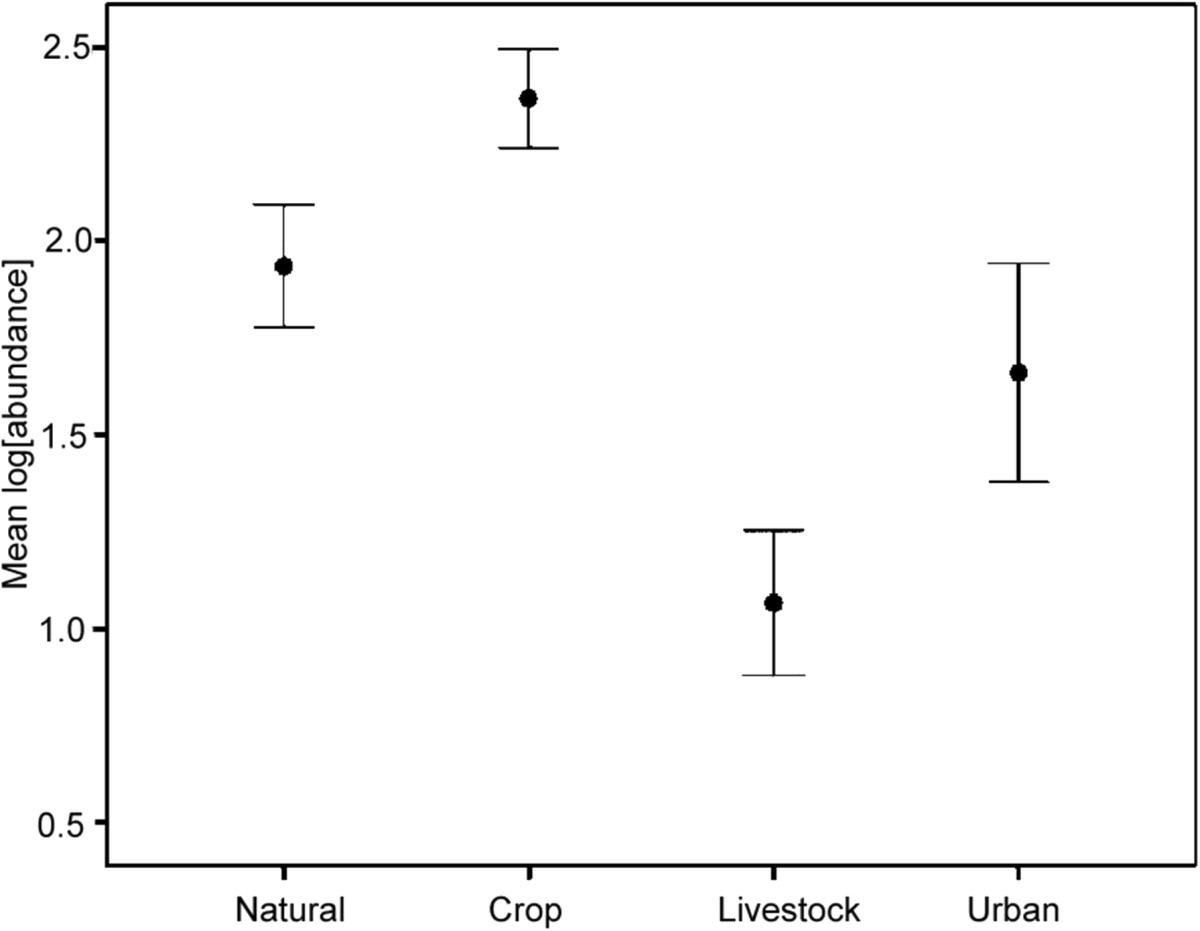
**Overall mean abundance (log[abundance]) of helminth species in** ***Rhabdomys pumilio*** **per habitat type.**

Eight helminth species were recovered (*Heligmonina spira, Neoheligmonella capensis, Trichostrongylus probulurus, Syphacia* sp.*, Trichuris* sp.*, Hymenolepis nana, Hymenolepis microstoma* and an unidentified trematode species). The overall helminth prevalence from 518 dissected host individuals was 86.68% (Figure 3). The nematode *Heligmonina spira* was with 77.22% the most prevalent worm. Animals were infected with one (22.01%), two (22.39%), three (23.94%), four (13.71%), five (4.05%), six (0.58%) or zero (13.32%) helminth species. Mean species richness was significantly higher in crop fragments compared to natural areas (Figure 4; Tables 2 and3), while it was significantly lower at livestock fragments and within urban areas. The overall helminth prevalence and abundance (pattern for abundance shown in Figure 5) showed similar results with significantly higher abundances in rodents trapped in crop fragments compared to the other three landscape types. Significantly lower prevalence values were recorded in livestock fragments and urban areas compared to natural landscapes. The most prevalent nematodes, *H. spira* and *N. capensis* revealed the same significant trends of higher prevalence and abundance at crop fragments and were less prevalent and abundant at livestock and urban areas. The pinworm *Syphacia* sp. was less prevalent at livestock and urban landscapes but its abundance was not significantly influenced by any landscape type. Prevalence and abundance of *T. probulurus* were lower at livestock farms and urban areas, while cestode prevalence (*Hymenolepis nana* and *H. microstoma* combined) did not show significant effects due to landscape type. However, tendencies of elevated prevalence at livestock and urban sites were recorded (Tables 2 and3). Whenever significant differences in infestation pattern between male and female hosts were discovered, males appeared to be more infected than females. Furthermore, parasite infections of all helminthes species as well as the parasite species richness were positively related to host length (Table 2).

**Table 2:** **Effect of different landscape types and host traits on helminth burden**

Response variable	Predictor	Coefficient ± SE	***t / z***	p	Effect	%DE
Species richness	Crop	1.72 ± 0.32	*t* = 5.330	<0.001	+	39.70
	Livestock	-1.14 ± 0.27	*t* = -4.300	<0.001	-	
	Urban	-1.55 ± 0.23	*t* = -6.768	<0.001	-	
	Length	0.18 ± 0.03	*t* = 6.950	<0.001	+	
	Sex	-0.38 ± 0.10	*t* = -3.831	<0.001	+ ♂	
	Density	-0.13 ± 0.03	*t* = -4.188	<0.001	-	
	Year 2004	0.94 ± 0.16	*t* = 5.860	<0.001	+	
	Year 2010	1.33 ± 0.20	*t* = 6.764	<0.001	+	
	Crop: Density	-0.17 ± 0.06	*t* = -2.971	0.003	-	
	Urban: Density	0.26 ± 0.05	*t* = 5.020	<0.001	+	
Helminth prevalence	Crop	1.46 ± 0.56	*z* = 2.604	0.009	+	33.05
	Livestock	-2.45 ± 0.47	*z* = -5.191	<0.001	-	
	Urban	-1.74 ± 0.50	*z* = -3.477	0.001	-	
	Length	0.33 ± 0.09	*z* = 3.894	<0.001	+	
	Density	-0.18 ± 0.07	*z* = -2.656	<0.001	-	
	Year 2010	3.39 ± 0.53	*z* = 6.347	<0.001	+	
Helminth abundance	Crop	1.02 ± 0.15	*t* = 6.762	<0.001	+	51.52
	Livestock	-1.44 ± 0.20	*t* = -7.245	<0.001	-	
	Urban	-1.70 ± 0.16	*t* = -10.503	<0.001	-	
	Length	0.06 ± 0.01	*t* = 5.331	<0.001	+	
	Density	-0.06 ± 0.01	*t* = -4.472	<0.001	-	
	Year 2004	0.39 ± 0.06	*t* = 6.046	<0.001	+	
	Year 2010	1.55 ± 0.15	*t* = 10.331	<0.001	+	
	Crop: Density	-0.12 ± 0.03	*t* = -4.341	<0.001	-	
	Urban: Density	0.14 ± 0.02	*t* = 6.081	<0.001	+	
*H. spira* prevalence	Crop	1.97 ± 0.55	*z* = 3.601	<0.001	+	47.87
	Livestock	-6.55 ± 1.06	*z* = -6.196	<0.001	-	
	Urban	-2.38 ± 0.47	*z* = -5.080	<0.001	-	
	Length	0.20 ± 0.08	*z* = 2.614	0.009	+	
	Density	-0.28 ± 0.11	*z* = -2.603	0.009	-	
	Year 2004	1.56 ± 0.74	*z* = 2.108	0.035	+	
	Year 2010	5.51 ± 0.98	*z* = 5.635	<0.001	+	
*H. spira* abundance	Crop	1.15 ± 0.16	*t* = 7.057	<0.001	+	53.81
	Livestock	-1.67 ± 0.23	*t* = -7.218	<0.001	-	
	Urban	-1.91 ± 0.16	*t* = -12.055	<0.001	-	
	Length	0.08 ± 0.01	*t* = 6.027	<0.001	+	
	Density	-0.07 ± 0.02	*t* = -4.266	<0.001	-	
	Year 2004	0.46 ± 0.08	*t* = 6.077	<0.001	+	
	Year 2010	2.18 ± 0.16	*t* = 13.926	<0.001	+	
	Crop: Density	-0.14 ± 0.03	*t* = -4.643	<0.001	-	
	Livestock: Density	-0.75 ± 0.14	*t* = -5.289	<0.001	-	
	Urban: Density	0.10 ± 0.03	*t* = 3.972	<0.001	+	
*N. capensis* prevalence	Crop	3.94 ± 0.69	*z* = 5.717	<0.001	+	26.13
	Livestock	-1.74 ± 0.65	*z* = -2.686	0.007	-	
	Urban	-2.56 ± 0.57	*z* = -4.459	<0.001	-	
	Sex	-0.85 ± 0.23	*z* = -3.777	<0.001	+ ♂	
	Length	0.25 ± 0.06	*z* = 4.351	<0.001	+	
	Density	-0.33 ± 0.07	*z* = -4.554	<0.001	-	
	Year 2004	1.79 ± 0.38	*z* = 4.768	<0.001	+	
	Year 2010	2.88 ± 0.49	*z* = 5.925	<0.001	+	
	Crop: Density	-0.40 ± 0.12	*z* = -3.364	<0.001	-	
	Livestock: Density	0.35 ± 0.14	*z* = 2.427	0.015	+	
	Urban: Density	0.46 ± 0.11	*z* = 4.019	<0.001	+	
*N. capensis* abundance	Crop	1.73 ± 0.33	*t* = 5.168	<0.001	+	30.57
	Livestock	-1.20 ± 0.41	*t* = -2.927	0.004	-	
	Urban	-1.95 ± 0.36	*t* = -5.359	<0.001	-	
	Sex	-0.26 ± 0.09	*t* = -2.827	0.005	+ ♂	
	Length	0.12 ± 0.02	*t* = 5.681	<0.001	+	
	Density	-0.13 ± 0.03	*t* = -3.708	<0.001	-	
	Year 2004	0.62 ± 0.13	*t* = 4.622	<0.001	+	
	Year 2010	1.90 ± 0.32	*t* = 6.019	<0.001	+	
	Crop: Density	-0.17 ± 0.07	*t* = -2.655	0.008	-	
	Urban: Density	0.20 ± 0.05	*t* = 3.970	<0.001	+	
*Syphacia* sp. prevalence	Livestock	-0.85 ± 0.39	*z* = -2.166	0.030	-	4.60
	Urban	-0.83 ± 0.40	*z* = -2.050	0.040	-	
	Sex	-0.44 ± 0.20	*z* = -2.195	0.028	+ ♂	
	Length	0.16 ± 0.05	*z* = 3.166	0.002	+	
	Year 2010	0.78 ± 0.36	*z* = 2.202	0.028	+	
*Syphacia* sp. abundance	Sex	-0.43 ± 0.17	*t* = 0.0123	0.012	+ ♂	1.79
*T. probolurus* prevalence	Livestock	-1.35 ± 0.37	*z* = -3.624	<0.001	-	12.85
	Urban	-1.70 ± 0.42	*z* = -4.056	<0.001	-	
	Length	0.27 ± 0.06	*z* = 4.704	<0.001	+	
*T. probolurus* abundance	Livestock	-1.57 ± 0.41	*t* = -3.806	<0.001	-	22.27
	Urban	-1.55 ± 0.41	*t* = -3.757	<0.001	-	
	Length	0.26 ± 0.04	*t* = 5.752	<0.001	+	
*Hymenolepis* sp. prevalence	Livestock	0.57 ± 0.34	*z* = 1.657	(0.098)	+	6.87
	Urban	0.57 ± 0.34	*z* = 1.657	(0.098)	+	
	Density	0.13 ± 0.04	*z* = 3.377	<0.001	+	
	Length	0.25 ± 0.06	*z* = 4.104	<0.001	+	

Generalized linear model results that showed a significant relationship between landscape type (crop, livestock, urban and natural habitat), host density, host length and host sex on species richness and helminth parasite prevalence and abundance in *R. pumilio*. Coefficients ± standard errors; *t* = t-value; *z* = z-value; p = significance value 0.05, values under 0.1 given in brackets; effect: + = increasing effect, - = decreasing effect, compared to natural habitat; %DE = percentage of explained deviance; ♂ = male individuals.

**Table 3:** **Helminth species and prevalence [%] recorded in** ***Rhabdomys pumilio*** **per landscape type**

Family and species	Natural	Crop	Livestock	Urban
Heligmonellidae				
*H. spira*	86.8%	94.2%	34.6%	68.1%
*N. capensis*	47.7%	64.2%	58.5%	45.7%
Oxyuridae				
*Syphacia* sp.	35.1%	35.2%	34.6%	31.8%
Trichostrongylidae				
*T. probolurus*	31.0%	40.0%	9.9%	6.7%
Trichuridae				
*Trichuris* sp.	1.4%	0.6%	0.0%	1.1%
Hymenolepididae				
*Hymenolepis* sp.	14.8%	25.4%	20.1%	24.7%

## Discussion

Evident from this study is the fact that landscape characteristics influence host and parasite richness, abundance and prevalence. In particular, crop fragments proved to be more favourable to rodents (larger body size) and helminths (higher species richness and burdens). In contrast, rodents were smaller and helminths were fewer (number of species and mean abundance) in fragments that were associated with cattle farming. In addition, rodents that occurred in landscapes that were associated with human linked landuse were more commonly infected with cestodes compared to extensive nature reserves.

We first elaborate on the effects of different habitat usage on the host and its helminth burden and second, assess the potential of recovered helminths and respective implications to act as zoonotic parasites.

### Human linked landscapes and gastro-intestinal helminth burdens

It is a well-known fact that economic development and human population growth go hand-in-hand with habitat transformation. The consequence is often a reduction in patch size and a change in natural plant communities and structure[21, 71]. This pattern is confirmed in the present study where crop, livestock and urban fragments were on average 100 times smaller compared to extensive natural areas. Vegetation structure and the surrounding matrix differed for each of the three fragment types. The vegetation structure in crop fragments was complex and should rather be referred as ground cover as it comprised a combination of remnant natural fynbos with patches of renosterveld vegetation (medium to large shrubs), chopped vegetation and timber logs that were left in the fragments. There was also a regular supply of water and seasonal wheat that grew on the fields or amongst the grape vines. In contrast, urban fragments comprised medium to high shrubs and trees and rodents were dependent on the surrounding matrix (urban gardens) for food and water, while livestock fragments consisted of low vegetation amongst open grassland. *Rhabdomys pumilio* is an opportunistic peri-domestic rodent[44] and dominated the rodent community in the four habitat types. Evident from the study was the fact that the landscape characteristics of crop fragments provided favourable conditions for the host as higher densities and larger body sizes were recorded. This pattern concurs with previous studies on *R. pumilio* in the South Africa[27, 28] and on California voles in the USA (*Microtis californicus*)[72]. Vegetation cover influences the presence and density of rodents[73–77] as it often provides resources such as shelter, food and nesting sites. Conducive microclimatic conditions in addition to larger and better quality hosts are possibly the drivers for the higher helminth species richness and larger infestations recorded in *R. pumilio* in crop fragments[28]. This pattern was also recorded for the two most abundant nematode species (*H. spira* and *N. capensis*). Belonging to the superfamily Trichostrongyloidea, *H. spira* and *N. capensis* are directly transmitted nematode species, with eggs and larval stages that occur in the external environment[78]. Vegetation structure (plant cover, life forms and height) not only affects terrestrial hosts, but also has a direct impact on parasite burdens as specific environmental conditions are necessary for egg and larval survival[79, 80]. Hulbert and Boag[13] investigated the role of habitat on intestinal helminths of mountain hares and recorded a larger parasite burden in woodland compared to open moorland. Moreover, relative humidity is generally higher within sheltered habitats than in more exposed sites[81], which directly benefits free-living stages[82]. More specifically, a recent study conducted across a natural precipitation gradient, revealed strong positive relationships between rainfall and humidity and helminth species richness as well as nematode abundance in *R. pumilio*[16]*.* In addition to environmental factors, host factors such as density and body size are often positively associated with parasite infestation levels. High host density can facilitate transmission of directly transmitted parasites, such as nematodes[28, 83, 84] while larger hosts are able to harbour larger parasite burdens and are often older, thus they had more time to accumulate permanent parasites such as helminths[28, 85, 86]. Given the above it is possible that crop fragments have an intermediate level of disturbance, which is facilitative of parasite communities[84–89]. Several studies have recorded this pattern for parasites[21, 90]. In particular, Friggens and Beier[91] confirmed this pattern for fleas and noted that agricultural systems that are resource-rich may counteract the negative response, by hosts and certain parasite species, to disturbance.

*Rhabdomys pumilio* individuals were smaller and present at lower densities in livestock and urban fragments compared to crop fragments. Disturbances within and surrounding the fragments may facilitate this pattern. Both former fragments were surrounded by less favourable matrixes due to the presence of large herbivores and domestic animals (cats and dogs). In addition, in urban fragments ranging behaviour of rodents was possibly limited to those areas of cover due to a high presence of domestic animals (cats and dogs), which may cause regular disturbances to foraging rodents. Limited range size may also play a role in livestock fragments as any movement by rodents outside the protection of cover may expose them to predation by raptors. In addition, the presence of livestock and high grazing intensity by cattle or sheep can negatively affect small mammals[92, 93]. Large herbivores (wild and domestic) consume the same vegetation as many rodents and they therefore have the potential to compete for food resources[93, 94]. They also reduce vegetation height and cover through trampling and grazing[95], which may damage nests and increase the exposure of small mammals to predation[96, 97]. The factors mention above can all contribute in concert to lower helminth species richness and infestation levels in the two latter landscapes. This pattern was recorded for the most abundant nematode species. These findings support a previous study conducted in Thailand where reduced helminth infections were recorded in rodents trapped amongst houses and villages[98]. Physical disturbance through trampling of soil may also provide a more direct negative effect on infective free-living stage of soil transmitted nematodes such as *Trichostrongylus* sp.

Parasite life cycle, transmission mode and behavior can influence the response to host and environmental factors[28, 99, 100]. The nematode *Syphacia* sp*.* was the third most abundant helminth species recorded in *R. pumilio*. No significant relationship was recorded between landscape type and abundance for this species. This pattern may be due to the fact that this nematode is less dependent on the external environment and host density as transmission of eggs are mainly through self-infection and direct body contact[28, 101, 102]. A similar relationship was recorded between host density and body-transmitted parasites, such as sucking lice on rodents[28] and wing mites on bats, compared to parasites that have free-living stages[103]. The cestodes *Hymenolepis nana* and *H. microstoma* were present at higher prevalence, albeit not significant, in livestock and urban fragments. The study by Chaisiri et al.[98] also recorded a positive relationship between *H. nana* in rodents and human linked landscapes such as villages and irrigated rice fields in Thailand. *Hymenolepis nana* does not require any intermediate host and therefore can be spread directly from host-to-host or as an autoinfection[104]. A common intermediate host of *H. microstoma* is e.g. the confused flour beetle (*Tribolium confusum*) that is a pest insect of damaged grain (often associated with livestock landscapes) and house-hold grain products (common in urban areas)[105].

### Helminths species and their potential to act as zoonotic parasites

The two most prevalent helminth species, *H. spira* and *N. capensis* (subfamily Nippostrongylinae) were previously recorded from *R. pumilio*[16, 106]. The exact life cycles of both species are still unknown but belonging to the superfamily Trichostrongyloidea, the cycles most probably include free-living larval stages. Both species were most prevalent and abundant in rodents trapped in crop fragments. Given the specific fragment characteristics it is likely that these species thrive best in humid conditions with plenty of vegetation structure. This pattern is supported by Froeschke et al.[16] where higher prevalence and infection intensity were recorded in areas with high precipitation and humidity. Members of the Nippostrongylinae are common in Muridae and widespread over the world but to our knowledge, so far, none of these two species have been recovered from non-rodent hosts.

The pinworm *Syphacia* sp. has been previously recorded in *R. pumilio*[16]. Pinworms are commensal oxyurid nematodes feeding on bacteria that inhabit the intestinal tract of many rodents*. Syphacia* sp. has a direct life cycle where the second larvae stage is protected within the egg capsule and gets passed on mainly through direct contact among host animals[101, 102]. The proximity to its host might be the reason why this nematode species did not show any significant associations in its abundance to landscape type[28]. Although nematodes from the family Oxyuridae also occur in domestic animals and humans, pinworms are usually non-pathogenic, even in large numbers[107]. However, they can cause rectal irritation and prolapse, lethargy, decreased weight gain and may influence the susceptibility of the host to other intestinal nematodes[108, 109].

Although *T. probulurus* is mainly known within ruminants [80| it has been previously detected in *R. pumilio*[16] and the Cape hare (*Lepus capensis*)[110]. Surprisingly the species was less prevalent and abundant in *R. pumilio* at livestock farms compared to natural localities in our study. Records of human infections with *Trichostrongylus* species are rare[111] but a recent study conducted on helminthiasis in Cape Town found *Trichostrongylus*-type eggs with a prevalence of 0.1% within school children[63]. *Trichostrongylus* species have a direct life cycle and humans are infected mainly through vegetable foods contaminated by infected animal droppings[112, 113]. Species from this genus have low clinical significance but in heavy infestations they may be able to cause blood loss in the host[111].

Whipworms of the genus *Trichuris* are common cosmopolitan nematodes and have also been recovered from *R. pumilio* before[16]. The species was present at low prevalence and abundance in *R. pumilio* in the present study. Nematodes of the genus *Trichuris* occur in several other Muridae species and eggs are transmitted by ingestion from soil[14, 114]. The biology of *Trichuris muris* is for instance very similar to the human whipworm, *Trichuris trichiura*, and the former is often used as model for the latter[87].

The dwarf tapeworm *Hymenolepis nana* has a worldwide distribution in rodents and humans[95, 105, 115]. Young mice are frequently infected and show weight loss, catarrhal diarrhea, focal enteritis, and death[116]. *Hymenolepis nana* is the only known cestode species which does not require an intermediate host but arthropods such as fleas among others can serve as such[105, 115]. Human infections have been reported from Algeria, Egypt, Sudan, Burkina Faso, Senegal and South Africa[115]. A study conducted in the Cape Town region by Adams et al.[63] revealed that eggs of the dwarf tapeworms were present (2.2% prevalence) in school children and it is possible that rats and dogs could be additional reservoir hosts. *Hymenolepis* eggs have been detected in faeces of dogs living together with their infected owners in Aboriginal communities in the north-west of Western Australia[117, 118]. The reservoir status of rats was confirmed in a recent study on helminth communities in *Rattus rattus* in Malaysia where a prevalence of 28.4% was recorded for *H. nana*[119]. Furthermore, we discovered the cestode *H. microstoma* with a prevalence of less than 2%. This species commonly infects mice[120, 121] and a recent study discovered this species for the first time in humans suggesting that it can possibly be regarded as a new zoonosis[122]. Eggs of *H. nana* and *H. microstoma* differ in size but are morphologically quite similar to each other and it is possible that *H. microstoma* in humans have previously been misdiagnosed as *H. nana*[123]. This is the first record of *Hymenolepis* species in *R. pumilio*.

We furthermore detected an unidentified digenea trematode species in low prevalence. Digeneans have a life-cycle involving at least 2 hosts, a definitive and 1 or 2 intermediate hosts[124]. Further investigation is necessary on its taxonomic classification and zoonotic potential but interestingly it was only recovered from hosts trapped in the three human linked habitats.

## Conclusion

To conclude, the helminth fauna of *R. pumilio* is diverse and includes both benign and zoonotic taxa. It is evident that resource-rich landscapes provide favorable conditions for diverse and abundant helminth infestations, while landscapes that are more closely associated with humans pose a larger risk through the presence of zoonotic species. Increased prevalence of zoonotic species in peri-urban landscapes is a concern given the fact that communities living in informal housing settlements often have poor sanitation and lack clear water. In addition, immuno-suppressive diseases, such as HIV-AIDS and tuberculosis, can predispose humans to helminth infestations, and vice versa. It is therefore essential that baseline data is established on the landscape characteristics and host species that pose a disease risk to domestic animals and humans. This endeavor will facilitate proactive surveillance for known and novel zoonotic diseases.

## Authors’ original submitted files for images

Below are the links to the authors’ original submitted files for images. Authors’ original file for figure 1 Authors’ original file for figure 2 Authors’ original file for figure 3 Authors’ original file for figure 4 Authors’ original file for figure 5
