# Supplementary figures and images for: Landscape characteristics influence helminth infestations in a peri-domestic rodent - implications for possible zoonotic disease
Source: Parasit Vectors. 2014 Aug 26;7:393. doi: 10.1186/1756-3305-7-393 (PMC4158073; doi:10.1186/1756-3305-7-393)

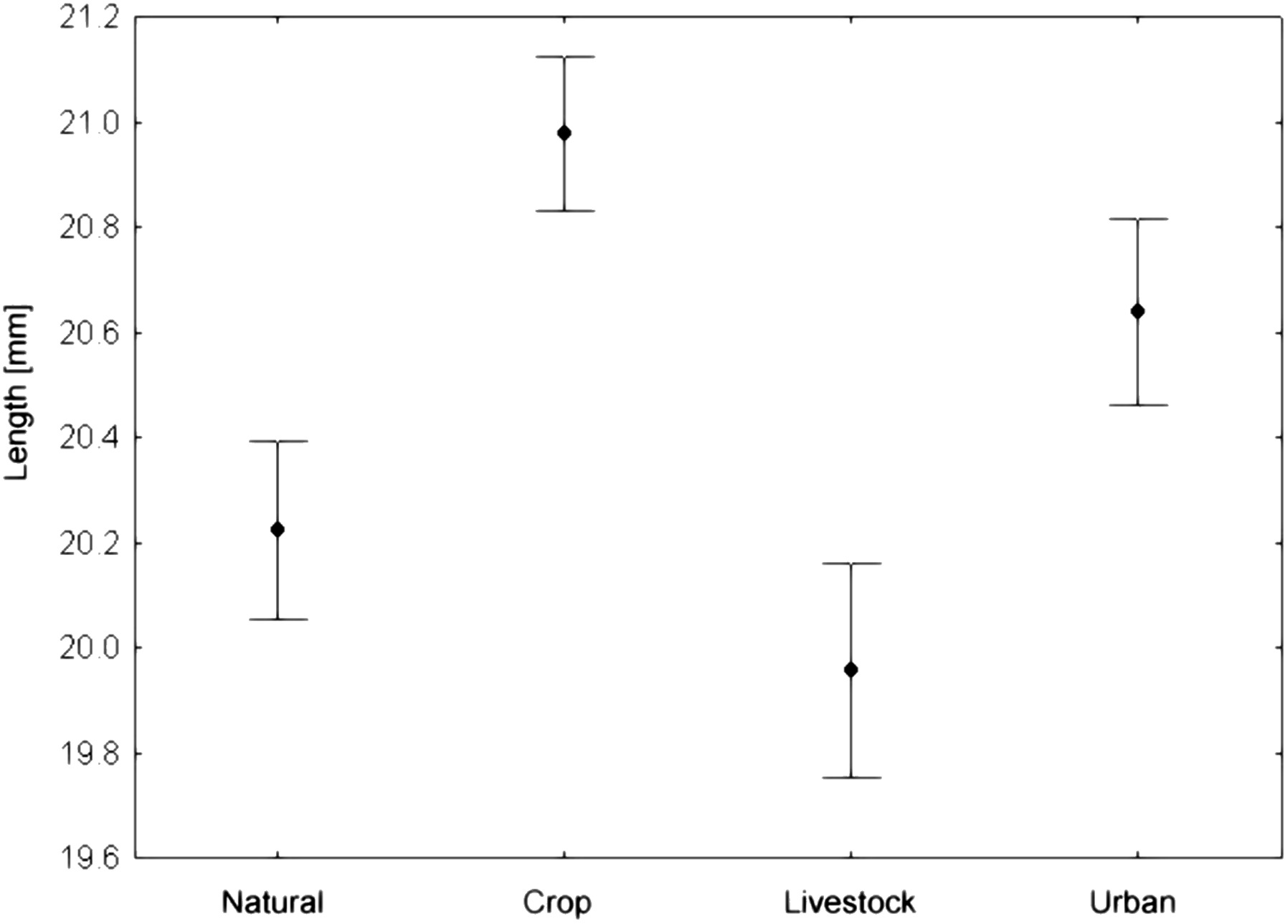

Supplement: Supplementary file 1 — Authors’ original file for figure 1 [file 13071_2014_1575_MOESM1_ESM.tif]

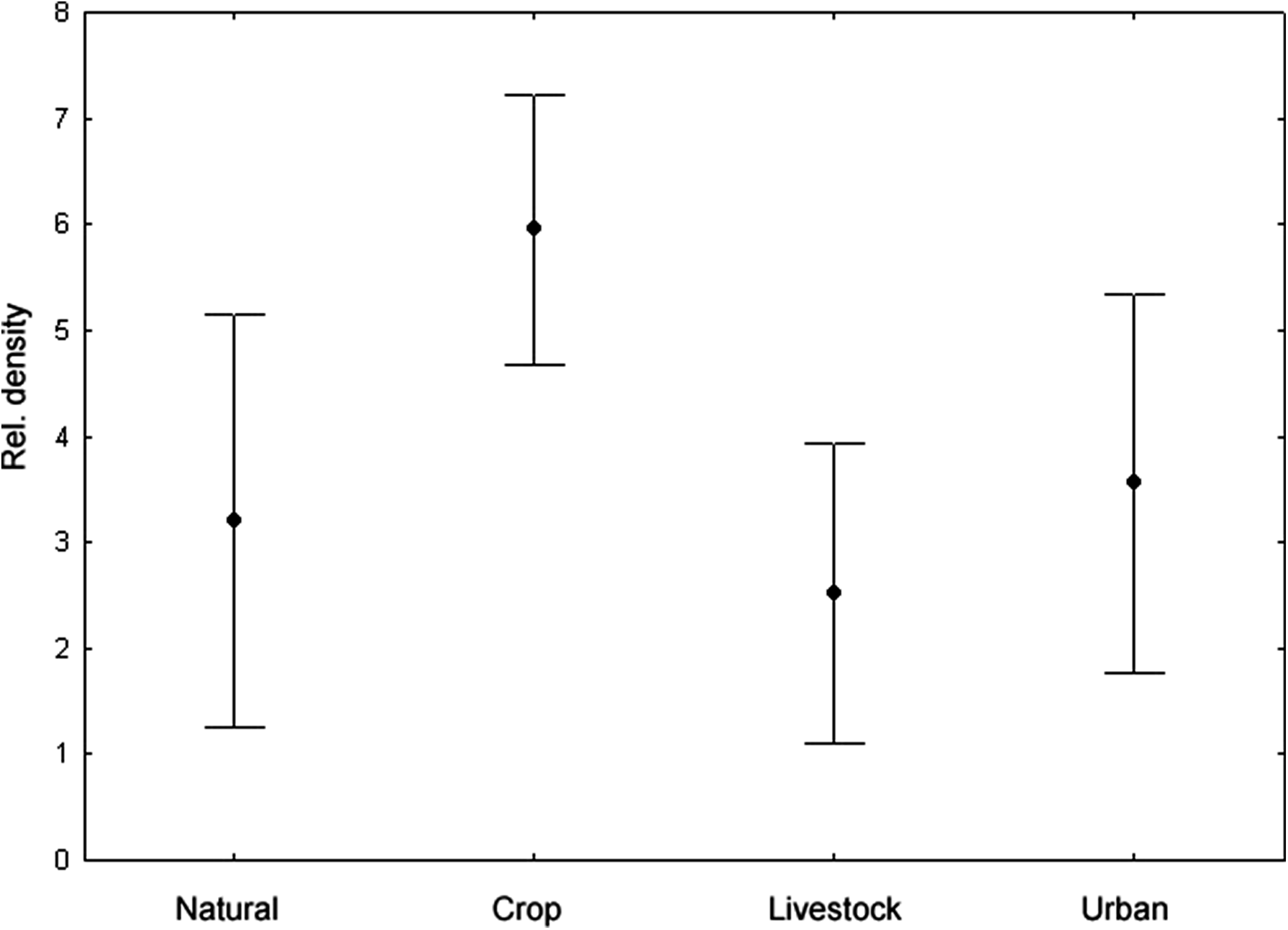

Supplement: Supplementary file 2 — Authors’ original file for figure 2 [file 13071_2014_1575_MOESM2_ESM.tif]

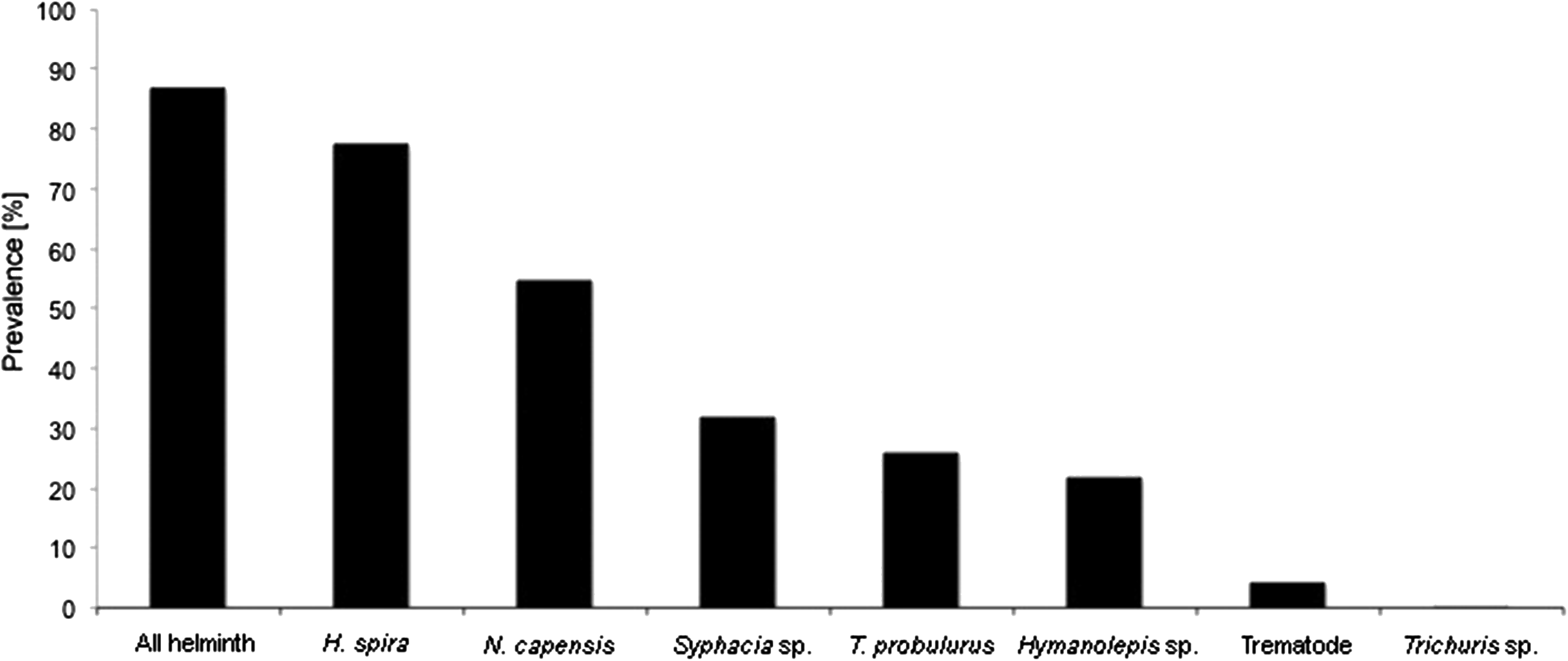

Supplement: Supplementary file 3 — Authors’ original file for figure 3 [file 13071_2014_1575_MOESM3_ESM.tif]

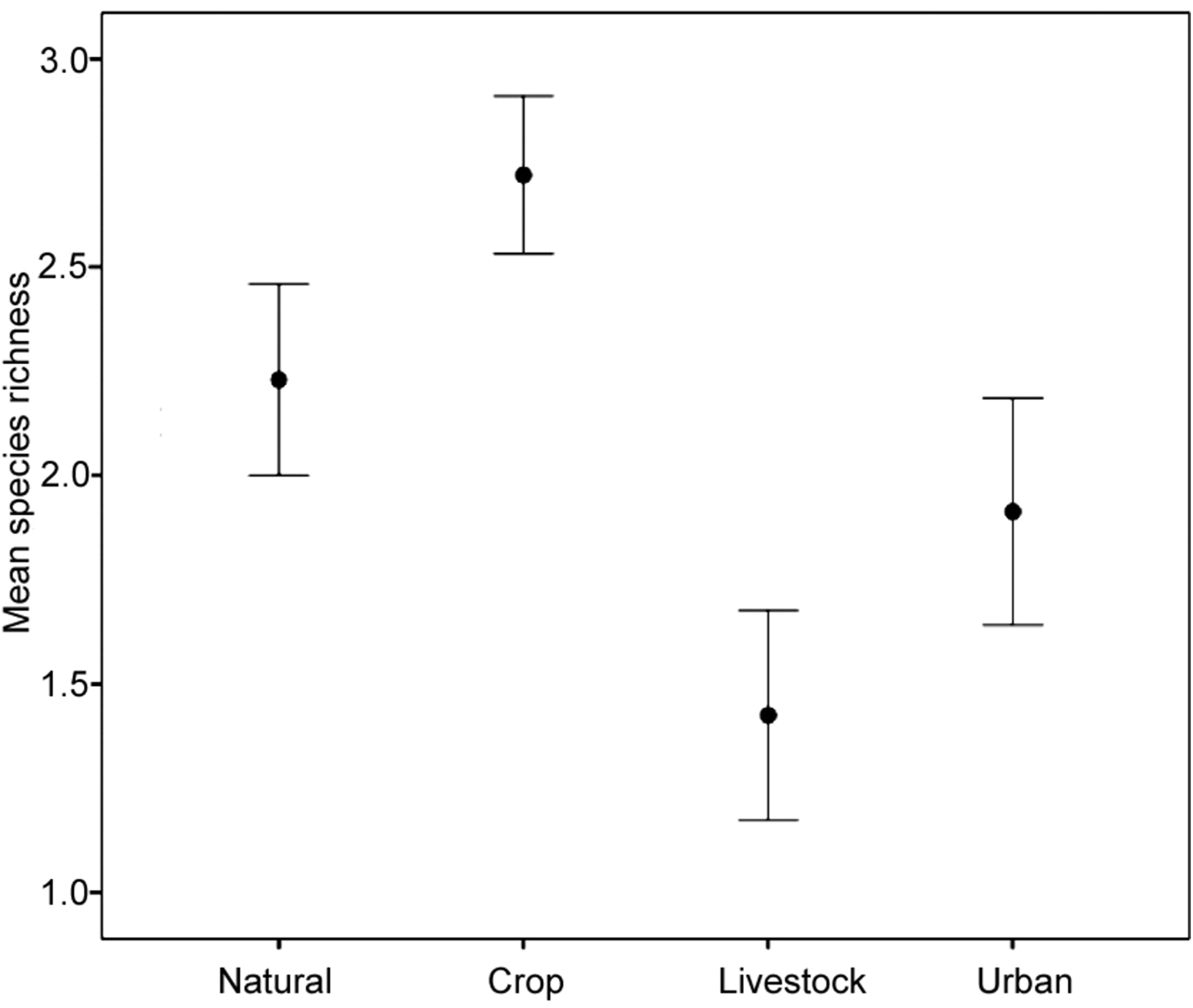

Supplement: Supplementary file 4 — Authors’ original file for figure 4 [file 13071_2014_1575_MOESM4_ESM.tif]

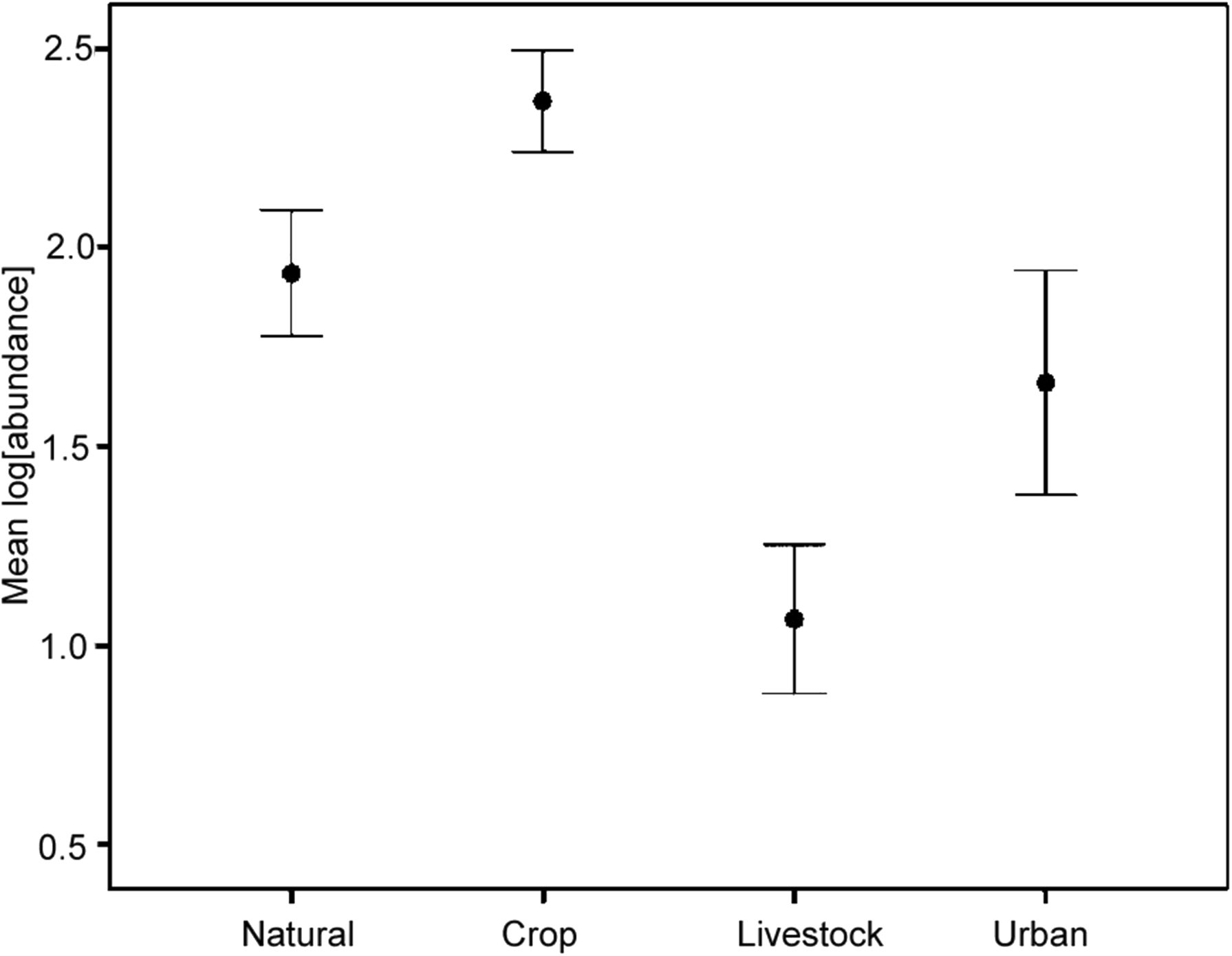

Supplement: Supplementary file 5 — Authors’ original file for figure 5 [file 13071_2014_1575_MOESM5_ESM.tif]
